# Global incidence and characteristics of spinal cord injury since 2000–2021: a systematic review and meta-analysis

**DOI:** 10.1186/s12916-024-03514-9

**Published:** 2024-07-08

**Authors:** Yubao Lu, Zhizhong Shang, Wei Zhang, Mao Pang, Xuchang Hu, Yu Dai, Ruoqi Shen, Yingjie Wu, Chenrui Liu, Ting Luo, Xin Wang, Bin Liu, Liangming Zhang, Limin Rong

**Affiliations:** 1 Department of Spine Surgery, The Third Affiliated Hospital, Sun Yat-Sen University, GuangzhouGuangdong, 510630 China; 2 Guangdong Provincial Center for Engineering and Technology Research of Minimally Invasive Spine Surgery, Guangzhou, 510630 Guangdong China; 3 Guangdong Provincial Center for Quality Control of Minimally Invasive Spine Surgery, Guangzhou, 510630 Guangdong China; 4 Department of Orthopaedics, The First Clinical Medical College of Lanzhou University, Lanzhou, 730000 Gansu China; 5 Department of Orthopaedics, Lanzhou University Second Hospital, Lanzhou, 730000 Gansu China

**Keywords:** Spinal cord injury, Incidence, Features, Systematic review, Meta-analysis

## Abstract

**Background:**

This study employs systematic review and meta-analysis to explore the incidence and characteristics of spinal cord injury (SCI) between 2000 and 2021, aiming to provide the most recent and comprehensive data support for the prevention, diagnosis, treatment, and care of SCI.

**Methods:**

Systematic searches were conducted on epidemiological studies of SCI published between January 1, 2000, and March 29, 2024. Meta-analysis, subgroup analysis, meta-regression, publication bias detection, and literature quality assessment were extensively utilized.

**Results:**

The pooled results from 229 studies indicated that the overall incidence rate of SCI was 23.77 (95% CI, 21.50–26.15) per million people, with traumatic spinal cord injuries (TSCI) at a rate of 26.48 (95% CI, 24.15–28.93) per million people, and non-traumatic spinal cord injuries (NTSCI) at a rate of 17.93 (95% CI, 13.30-23.26) per million people. The incidence of TSCI exhibited a marked age-related increase and was significantly higher in community settings compared to hospital and database sources. Males experienced TSCI at a rate 3.2 times higher than females. Between 2000 and 2021, the incidence of TSCI remained consistently high, between 20 and 45 per million people, whereas NTSCI incidence has seen a steady rise since 2007, stabilizing at a high rate of 25–35 per million people. Additionally, the incidence of TSCI in developing countries was notably higher than that in developed countries. There were significant differences in the causes of injury, severity, injury segments, gender, and age distribution among the TSCI and NTSCI populations, but the proportion of male patients was much higher than that of female patients. Moreover, study quality, country type, and SCI type contributed to the heterogeneity in the meta-analysis.

**Conclusions:**

The incidence rates of different types of SCI remain high, and the demographic distribution of SCI patients is changing, indicating a serious disease burden on healthcare systems and affected populations. These findings underscore the necessity of adopting targeted preventive, therapeutic, and rehabilitative measures based on the incidence and characteristics of SCI.

**Supplementary Information:**

The online version contains supplementary material available at 10.1186/s12916-024-03514-9.

## Background

Spinal cord injury (SCI) results in a significant disease burden and severe public health issue due to its notably high disability rate, serious complications, limited treatment options, and substantial medical expenses [1–7]. Hence, proactive prevention and effective care are of utmost importance. Preventing SCI through epidemiological research is an urgent yet practical strategy. Epidemiological data form the foundation for tracking the incidence of SCI, formulating prevention and diagnosis strategies and planning the allocation of medical resources [8, 9]. Epidemiological surveys provide insights into the incidence, injury mechanisms, and patient characteristics of SCI in different scenarios. This enables the development of targeted prevention guidance and scientifically standardized treatment processes. This ensures that patients receive timely and effective treatment, reducing the incidence of secondary injuries and serious complications. It also serves as a reference for constructing animal models (species, gender, age, etc.) and designing experimental details (injury type, affected segments, outcome measurement, reporting, etc.) [10].

The reported incidence of SCI varies significantly, ranging from 7 [11] to 152.2 per million people [12]. Moreover, most research is primarily concentrated in developed regions such as North America, Europe, and Australia, contrasting with the scant data available from Asia and other developing regions [13–15]. Conducting epidemiological surveys of SCI on a global scale is time-consuming and labor-intensive. Systematic reviews and meta-analyses (SRs/MAs) offer new possibilities. Although numerous SRs/MAs have explored the epidemiological status of SCI, the existing studies commonly exhibit apparent limitations. These include restricted databases for retrieval, insufficient time ranges, incomplete search terms, and lack of quality assessments and in-depth data analysis [16–19]. These factors greatly diminish the evidence quality and reliability of the outcomes of the SRs/MAs. Furthermore, these studies still rely on data published before the year 2000, which limits their value in guiding the current prevention and treatment of SCI. As an important central nervous system disorder, SCI urgently requires reliable and effective epidemiological data to inform disease management decisions. Thus, updated and comprehensive SRs/MAs are necessary. In this study, we systematically evaluate the available global data on the incidence and characteristics of SCI to elucidate the rates and demographic distributions of SCI under various conditions. We hope this work will enhance global awareness of SCI and provide a knowledge base for future public health policy formulation and clinical trial execution.

## Methods

This study adheres to the PRISMA 2020 guidelines [20] and was registered in advance on PROSPERO (CRD42023400230).

### Inclusion and exclusion criteria

We established rigorous inclusion and exclusion criteria based on the PICOS (participants, interventions, comparators, outcomes, and study design).

#### Patients (P)

Individuals of all ages, genders, and ethnicities diagnosed with SCI, including various types of SCI patients, without restriction to specific geographic locations.

#### Interventions (I)

Not applicable, as this systematic review focuses on observational studies primarily concerned with the incidence and characteristics of SCI.

#### Control (C)

Not applicable, since the main goal is descriptive analysis.

#### Outcome (O)

Includes studies reporting the incidence of SCI or those that can calculate the incidence of SCI. Research aimed at investigating or describing the characteristics of SCI patients, such as patient origins, gender ratios, age, location of injury, severity, and occupation, also meets the inclusion criteria.

#### Type of study (S)

Comprises observational studies (such as cohort studies, case-control studies, and cross-sectional studies) and descriptive studies.

#### Exclusion criteria

Studies that fail to report the incidence or patient characteristics of SCI, as well as those with incomplete data—including commentaries, case reports, editorials, guidelines, letters to the editor, and conference abstracts—will be excluded. Articles that conflate spinal fractures with SCI are also excluded, unless the SCI patient data are separately reported. Additionally, studies that amalgamate data from before and after the year 2000 are excluded. Studies reporting an average annual incidence over a period of 2 years or longer, where the data cannot be obtained or converted into an annual incidence rate, will be excluded.

### Data sources and searches

Assisted by an experienced librarian, we conducted searches for keywords associated with spinal cord injuries, including their incidence, etiology, and characteristics, utilizing both MeSH terms and free text for a comprehensive approach. We explored a range of databases for this research, specifically Web of Science, PubMed, Embase, Cochrane, Scopus, ProQuest, OpenGrey, National Technical Information Service, WHO International Clinical Trials Registry Platform, and the US National Institutes of Health database. The search covered the period from January 1, 2000, to February 16, 2023, with a follow-up search on March 29, 2024, to capture the latest research. A detailed search strategy can be found in Additional file 1: Table S1. We also searched the references of relevant studies for potential research.

### Literature screening and data extraction

The literature screening and information extraction were conducted by six researchers. This process utilized a paired cross-checking method, with a third individual assisting in resolving any divergent opinions about specific publications. Initially, we identified research that appeared to meet the inclusion criteria based on titles and abstracts. The full texts were then reviewed to confirm their eligibility, and relevant information was extracted using a predefined data extraction form. The primary information gathered included: (1) basic details: first author, publication year, study type, time, and location of the study, source, and number of patients and (2) outcome measures: incidence and characteristics of SCI (severity, injury site, etiology, etc.).

### Evidence quality assessment

The appraisal of evidence quality in the included studies was conducted using an evaluation tool developed by the Joanna Briggs Institute, which is specifically designed for epidemiological research. This tool assesses studies on various criteria, including the scale and representativeness of the population, methods of recruitment, standards for disease diagnosis, reliability of outcomes, methods of data analysis, and the management of confounding factors [9]. The assessment encompasses ten items, each scored as either 0 or 1. An individual study’s total score can range from 0 to 10, with scores ranging from 0 to 4 indicating low research quality, 5 to 7 indicating moderate quality, and 8 to 10 indicating high quality. Regarding each item, the studies are considered to have performed satisfactorily if 60% or more meet the specific criterion for that item, signifying that the included studies generally exhibit good performance in that area.

### Statistical analysis

Given the high anticipated heterogeneity among studies, a random effects model was employed for the meta-analysis. Statistical analysis was conducted using the Metaprop command in STATA. Metaprop is tailored for binomial data, capable of calculating exact binomial confidence intervals and test-based intervals for scores. It employs suitable methods for dealing with proportions where normal approximation is inadequate, using the binomial distribution to model within-study variability and the Freeman–Tukey double arcsine transformation for variance stabilization [21]. Heterogeneity is quantified using the I-squared measure, with values of 25% or lower indicating low heterogeneity, 26% to 50% suggesting moderate heterogeneity, and over 50% representing high heterogeneity [22, 23].

When compiling the incidence of SCI, we treated each year reported in a study as a separate entity if the study provided data for multiple years. Consequently, the number of studies included in the analysis does not match the actual number of published papers. Our analysis reports years based on the actual survey periods of the studies, rather than their publication years. This ensures that the incidence rates of SCI reported reflect the accurate years of occurrence. We excluded studies that only reported an average SCI incidence rate over multiple years without annual data, as they did not provide specific yearly incidence rates. When a study reported incidence rates that included data before the year 2000, we only extracted data from the year 2000 onwards. When studies reported incidence rates adjusted by gender or age, as well as crude incidence rates (or data that allowed us to calculate crude incidence rates), we utilized the crude incidence rates to facilitate comparisons across studies. We calculated the overall incidence rates of SCI, as well as for traumatic and NTSCI, separately, based on the type of injury. Incidence rates were calculated for different subgroups based on the country of the study, the year, data source (hospital, community, database), severity of SCI (American Spinal Injury Association Impairment Scale, AIS [24]), injury location (cervical, thoracic, lumbar, cervicothoracic, injuries involving three or more segments were defined as multi-segmental injuries), and the patient’s age and gender. In patients with SCI, we calculated the proportions of different subgroups based on the location and severity of the SCI, as well as the patient’s age, occupation, and the cause of the injury. We used Egger’s test to quantitatively assess publication bias, rather than funnel plot analysis, due to the subjective nature of assessing symmetry in funnel plots [25]. We employed univariate meta-regression models to evaluate the relationship between study characteristics and the overall incidence rates of SCI, aiming to identify the causes of heterogeneity.

## Results

### Study selection and characteristics

**Fig. 1:**
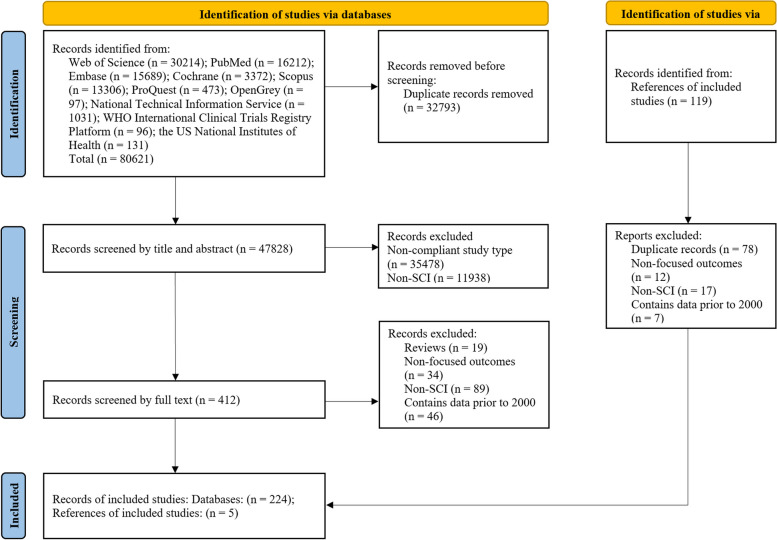
Flowchart of literature screening process

We retrieved 80,621 articles from 10 databases and obtained an additional 119 articles from reference lists. After removing duplicates and screening titles, abstracts, and then full texts, a total of 229 articles were ultimately included. The process of article selection is detailed in Fig. 1.

**Fig. 2:**
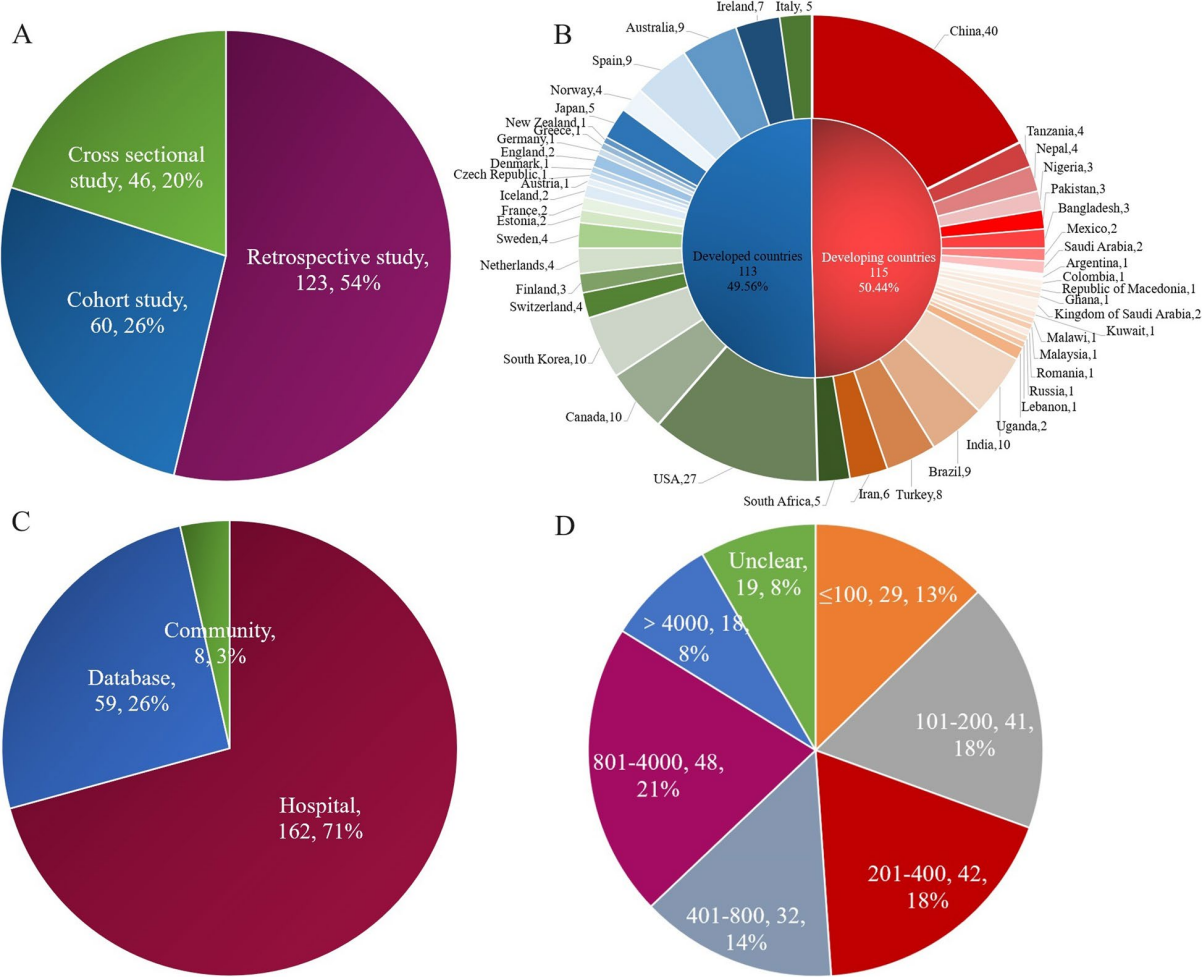
Basic information of included studies (category name, number of studies, proportion. **A** Type of study. **B** Number of publications by country. **C** Source of data. **D** Sample size of the studies included)

Out of the 229 studies, 60 were prospective, 46 were cross-sectional, and 123 were retrospective (Fig. 2A). These studies were conducted between 2000 and 2021. They originate from 47 countries, with the highest number from China (40 studies) and the USA (27 studies), followed by South Korea, Canada, and India, each contributing 10 studies (Fig. 2B). The sources of data included hospitals (162 studies), databases (59 studies), and communities (8 studies) (Fig. 2C). The total number of SCI patients was 531,299, with 19 studies not reporting the number of patients (Fig. 2D). Detailed information on the included studies can be found in Additional file 1: Table S2.

### Study quality

**Fig. 3:**
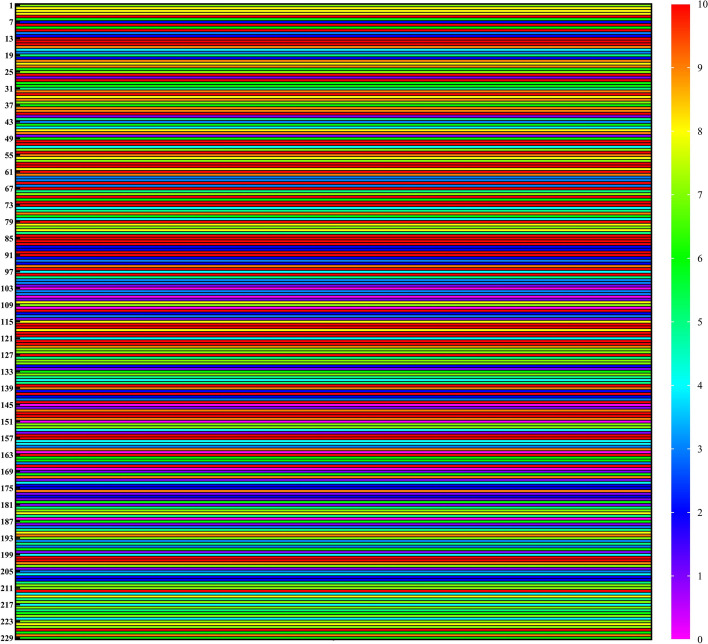
The quality of evidence in included studies (higher scores indicate higher quality of evidence in the studies)

The quality of 77 studies was low, 62 studies were of medium quality, and 90 studies were of high quality, as detailed in Fig. 3. The included studies were of lower quality in terms of the recruitment methods of participants, the comprehensiveness of data analysis, the criteria for determining SCI, and the identification, interpretation, and handling of confounding factors/subgroups.

### Incidence of SCI

**Fig. 4:**
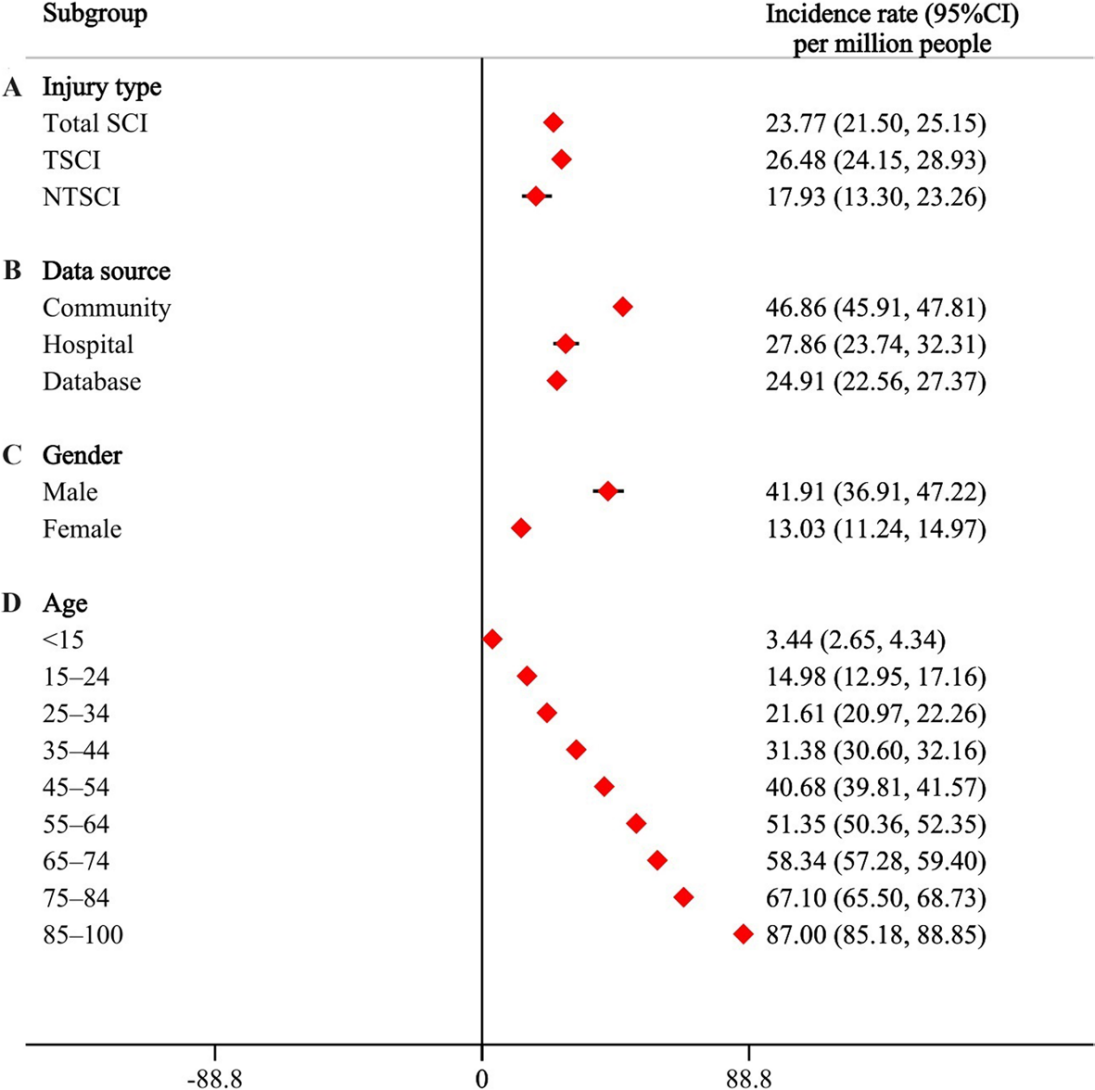
Incidence of spinal cord injury (**A** Injury type. **B** Data source. **C** Gender. **D** Age)

The incidence data of SCI were reported across 83 studies, encompassing a total of 324 sets of usable data. Analysis based on different types of SCI revealed an overall incidence rate of 23.77 (95% CI, 21.50–26.15) per million people. The incidence rate for TSCI was 26.48 (95% CI, 24.15–28.93) per million people, while the rate for NTSCI was 17.93 (95% CI, 13.30–23.26) per million people, as detailed in Fig. 4A. Due to the limited data on NTSCI, this study only calculated the total and annual incidence rates for NTSCI.

An analysis based on different data sources found that the incidence rate of TSCI from community surveys was as high as 46.86 (95% CI, 45.91–47.81) per million people, followed by those identified in hospitals and communities, as detailed in Fig. 4B.

Gender-based analysis revealed that the incidence rate of TSCI in females was 13.03 (95% CI, 11.24–14.97) per million people, while the rate in males was approximately 3.2 times higher, at 41.91 (95% CI, 36.91–47.22) per million people, as detailed in Fig. 4C.

Age-based analysis showed that the incidence rate of TSCI in individuals under 15 years of age was 3.44 (95% CI, 2.65–4.34) per million people. The incidence rate significantly increases with age, as detailed in Fig. 4D.

**Fig. 5:**
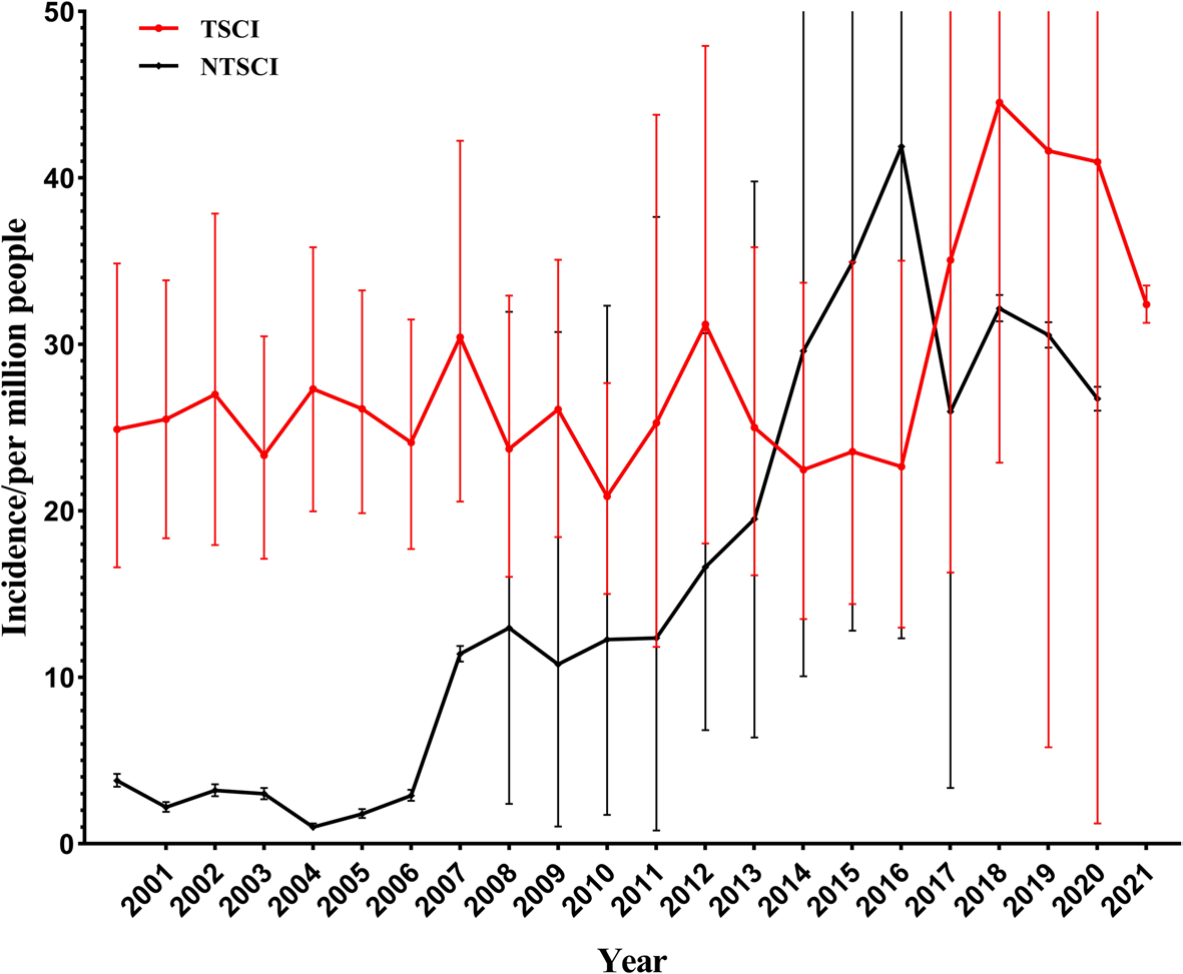
Annual incidence rates of spinal cord injury (the dots represent effect sizes, with the upper and lower edges corresponding to the upper and lower limits of the 95% CI)

Year-based analysis reveals that the incidence rate of TSCI has consistently remained at a relatively high level of 20–45 per million population. Specifically, the lowest incidence rate of TSCI was recorded in 2010, at 20.87 (95% confidence interval (CI), 15.01–27.68) per million population. From 2000 to 2016, the incidence rate of TSCI stabilized at below 30 per million population. In 2018, the incidence rate of TSCI peaked at 44.53 (95% CI, 22.89–73.31) per million population, followed by a decreasing trend, but it still maintained a relatively high level of above 30 per million population. Conversely, the incidence rate of NTSCI has gradually increased since 2007, maintaining a relatively high level of 25–35 per million population. Specifically, the incidence rate of NTSCI remained at a very low level (below 4 per million population) between 2000 and 2006, after which it increased annually, peaking in 2016 at 41.89 (95% CI, 12.35–88.92) per million population, followed by a decreasing trend, but it still remained above 25 per million population. For further details, see Fig. 5 and Additional file 1: Table S3 and Table S4.

**Fig. 6:**
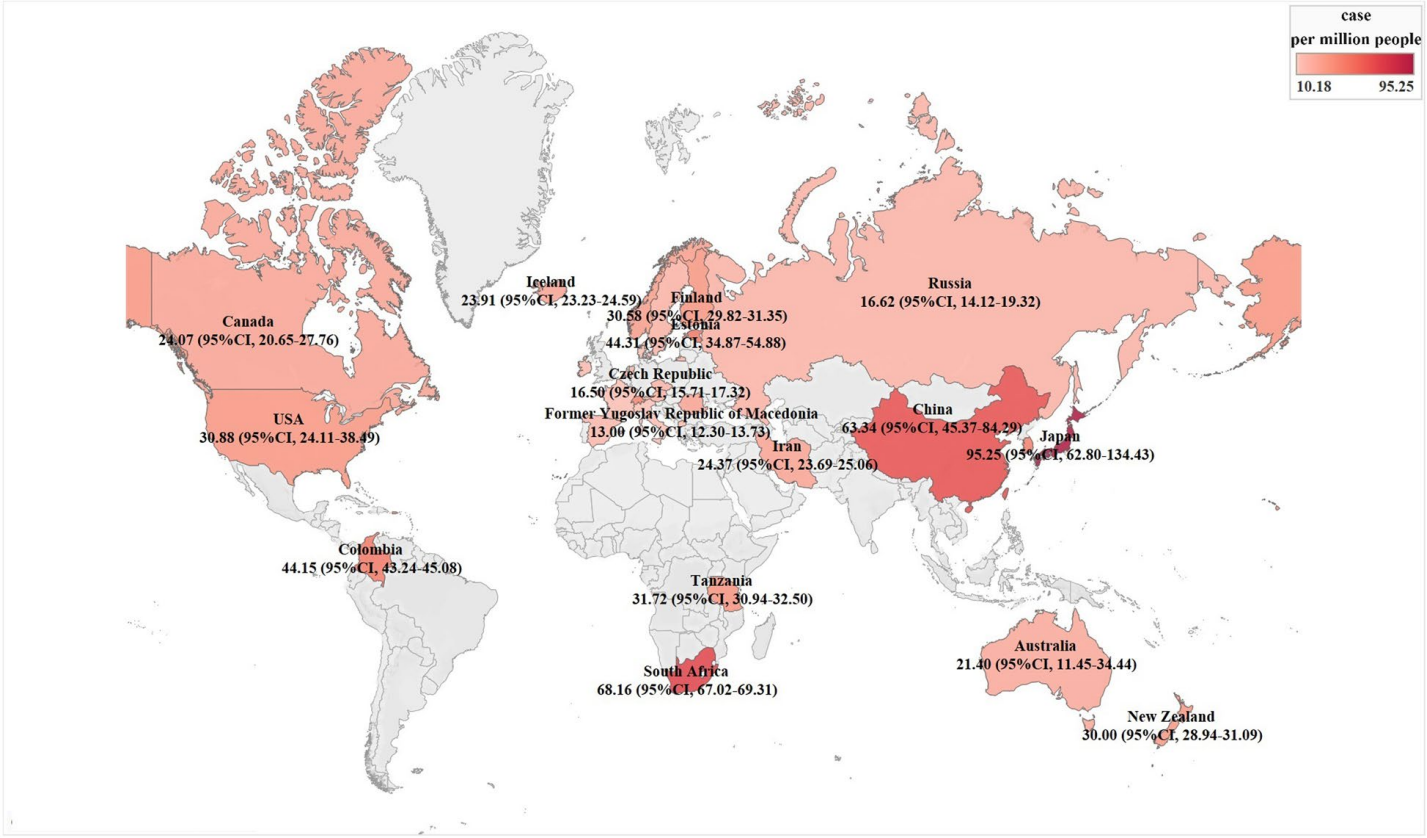
Incidence of spinal cord injury by country (darker colors indicate higher incidence rates)

Data was collected from 29 countries. In developed countries, the incidence rate of TSCI was 16.40 (95% CI, 16.20–16.60) per million, whereas in developing countries, it was 30.17 (95% CI, 29.82–30.53) per million. Notably, Japan reported the highest incidence rate of TSCI at 95.25 (95% CI, 62.80–134.43) per million, while Denmark reported the lowest at 10.18 (95% CI, 8.24–12.32) per million. Details of these findings are provided in Fig. 6 and Additional file 1: Table S5. Reports of data from countries in Africa and South America were less frequent.

Only one study has documented the incidence rates of TSCI by anatomical location, revealing rates of cervical injuries at 9.24 per million, thoracic injuries at 4.85 per million, and lumbar injuries at 1.64 per million [26]. Another study solely reported the incidence rate of cervical spinal injuries as 13.1 (95% CI, 11.4–14.9) per million [27].

Moreover, research on the incidence rates of SCI varying by severity levels or different occupations is currently nonexistent.

### Overview of SCI

**Fig. 7:**
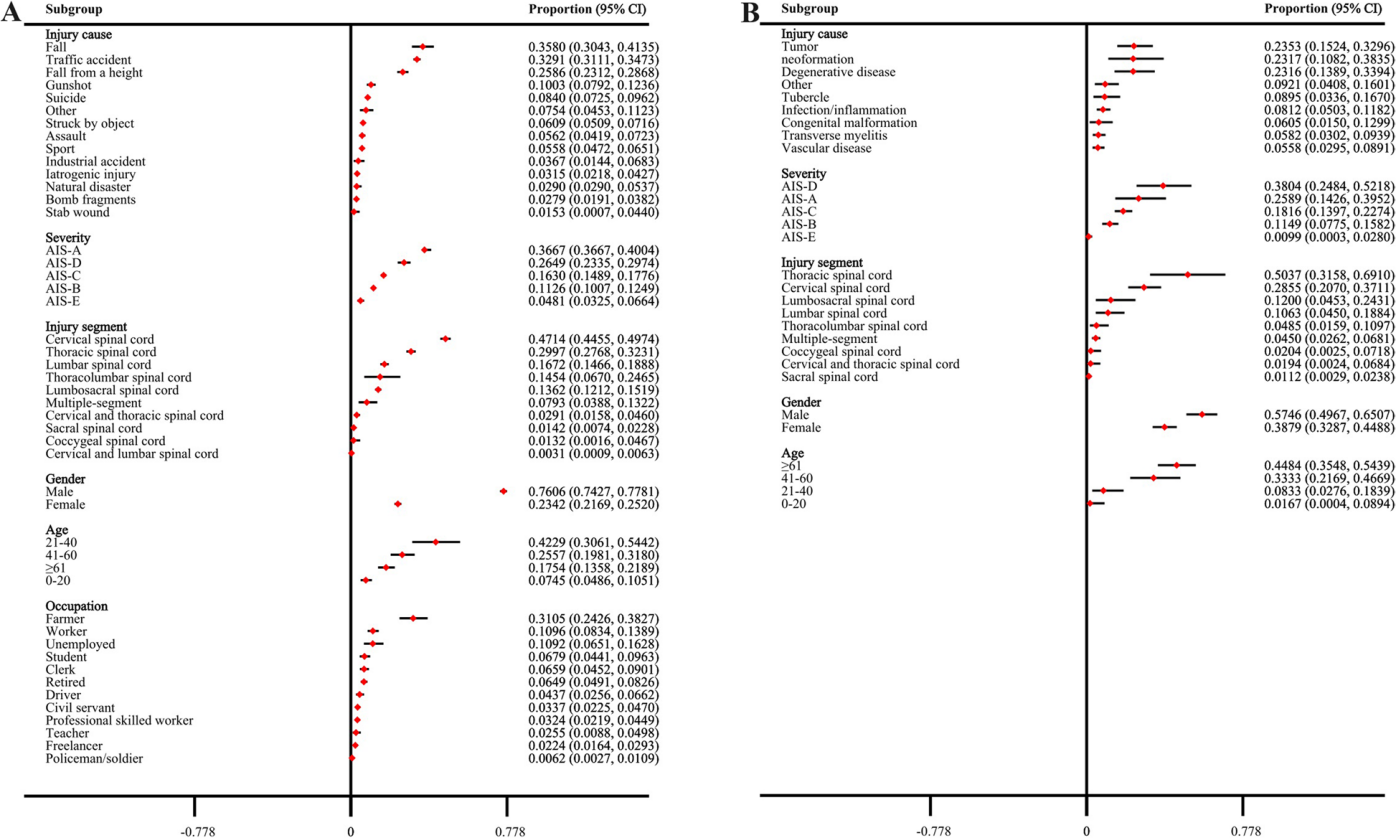
Distribution of population characteristics in spinal cord injury (**A** TSCI. **B** NTSCI)

In the population affected by TSCI, falls are identified as the predominant cause of injury. Conversely, tumors are attributed as the most frequent cause in the NTSCI cohort. Within the TSCI group, most patients are categorized under the AIS-A level, denoting the most severe impairment, whereas in the NTSCI group, a majority are classified at the AIS-D level, indicating a lesser degree of impairment. The cervical spine emerges as the most commonly injured site in TSCI patients, while the thoracic spine is more frequently affected in those with NTSCI. Moreover, in both TSCI and NTSCI groups, male patients significantly outnumber female ones. Among TSCI patients, the majority are aged 21 to 40 years, while in the NTSCI population, individuals over the age of 60 predominate. Farmers are reported as the most common occupation among TSCI patients, yet occupational data for NTSCI patients is still lacking in existing studies (Fig. 7).

### Heterogeneity and publication *bias*

Meta-regression analysis revealed that the quality of the studies, the type of country, and the type of SCI contributed to the heterogeneity in meta-analysis. However, the year of publication, the source of data, and the country were not associated with the heterogeneity observed in the meta-analysis, as detailed in Additional file 1: Table S6. The results of Egger’s test indicated a *t*-value of 47.66 with a *P*-value of less than 0.05, and a 95% CI for publication bias was [80.04, 86.93]. This suggests the presence of significant publication bias in the current studies.

## Discussion

This investigation aggregates the latest data to assess the epidemiologic characteristics of SCI from 2000 to 2021. Most current epidemiological studies are focused on TSCI, highlighting a significant research gap in NTSCI [28]. Based on limited data, this study found that although NTSCI incidence is lower than TSCI, it has been steadily increasing, suggesting NTSCI is gradually emerging as a significant category of SCI. Similar to previous findings, there has been no significant change in the overall global incidence of both SCI and TSCI [29]. However, the incidence of TSCI has varied from 20 to 45 per million, demonstrating a high level of persistence. This variability likely reflects the combined effects of multiple factors, including traffic accidents, falls, sports injuries, and the global trend towards an aging population. Particularly in 2010 and 2018, significant fluctuations in TSCI incidence highlight the impact of preventive measures, safety regulations, potential regional conflicts, and increases in traffic accidents. Concurrently, the incidence of NTSCI has exhibited an upward trend since 2007, likely associated with improvements in diagnostic techniques, an aging population, and an increase in chronic diseases. This trend reflects the challenges faced by healthcare systems in addressing such injuries. The low incidence rate of NTSCI from 2000 to 2006, followed by a rising trend, may reveal enhanced awareness of this type of injury, advancements in diagnostic technology, and improvements in data recording and reporting mechanisms. Further analysis of data sources indicates that the incidence of TSCI based on community surveys is nearly twice that of those based on hospital and database records. This higher incidence reflects numerous cases of SCI that may not be recorded or diagnosed by hospitals in the initial stages, particularly in areas with limited or may lack adequate medical facilities to accurately diagnose and report SCI [30]. Therefore, community surveys offer a closer approximation to the actual situation of data collection, capable of revealing cases that might be missing from hospital data. However, patients from the community may have been previously hospitalized and already counted as inpatients, leading to potential double-counting. This could result in the overestimation of the incidence rate of SCI in community surveys. Moreover, these findings underscore the importance of adopting preventive measures at the community level, as well as the implementation of community-based data management and provision of rehabilitation services to address the long-term rehabilitation needs of TSCI patients.

Contrary to previous studies which indicated that the relative frequencies of traffic accidents and falls as causes of TSCI were 43.18% and 34.24% respectively, our findings suggest that the etiological trends of TSCI have shifted, with the incidence of SCI due to falls now surpassing the numbers caused by traffic accidents [31]. Given the increasing severity of population aging, it is not surprising that the proportion of TSCI due to falls has been gradually increasing [32, 33]. Aging is also a contributing factor to the rise in the incidence of TSCI caused by degenerative diseases. Nonetheless, traffic accidents, as a primary cause of TSCI, cannot be overlooked. This necessitates an enhanced focus on fall prevention for the elderly in public health interventions, along with continued efforts to improve road traffic safety. Especially considering the global trend of aging populations, fall prevention should not only target the elderly but also include environmental modifications, increased physical activity, and improved bone health. Regarding traffic accidents, beyond traditional safety education and regulatory enforcement, new technologies such as autonomous driving assistance systems could also be considered to reduce the occurrence of accidents. In addition to the previously discussed causes, the etiology of TSCI is also influenced by lifestyle and political environments. For instance, the incidence of gunshot wounds and violence-related injuries in countries like Afghanistan [34] , Jordan [35], and Turkey [36] is significantly higher compared to other nations [37]. With the growing popularity of sports and the development of tourism, sports- and recreation-related TSCIs are becoming increasingly common worldwide, and specific sports are associated with a higher risk of TSCI [38]. The incidence of TSCI caused by sports such as diving, skiing, football, and horseback riding is increasing year by year [39]. In the realm of NTSCI, the prevalence of tumors, neoplasms, and degenerative diseases underscores the importance of early diagnosis and treatment of these conditions. Tumors and neoplasms can affect spinal cord function either by directly invading the spinal cord or by compressing it. Degenerative diseases, such as spinal stenosis and intervertebral disc degeneration, reflect natural physiological changes that occur with aging. The causes of TSCI and NTSCI span a broad spectrum, from external environmental factors to internal health conditions, highlighting the need for multifaceted prevention strategies that encompass improving public safety, promoting healthy lifestyles, and the early diagnosis and treatment of internal diseases.

Indeed, many countries lack sufficient evidence to demonstrate the epidemiological trends of SCI. The majority of available data originates from developed regions such as Europe, North America, and Australia, where systems for spinal injury registration or databases have been established, facilitating the collection of patient information. However, epidemiological research in some developing countries shares common shortcomings, often being hospital-based and failing to account for patients who die before reaching a hospital. Some individuals may opt to stay at home for self-treatment or seek alternative traditional therapies for various reasons. Despite the limited data from developing countries, the incidence of TSCI there is still significantly higher compared to developed countries. Moreover, the diagnostic techniques and systems for SCI in developing countries are not as advanced as those in developed countries, leading to a possibility that mild SCI may be overlooked [40]. Consequently, the incidence rate of SCI in developing countries might be underestimated. Currently, developing countries lack stringent registration systems and records of patients who die at scene of the injury or during the pre-hospital phase. This could explain why developed countries, which do register pre-hospital deaths, report higher incidence rates of SCI [41]. Notably, Japan’s incidence rate of TSCI is significantly higher than that of other countries, reaching 95.25 per million people. Previous studies have provided explanations for the high prevalence of SCI in Japan [42–46]. Firstly, Japan is one of the countries that is most severely affected by population aging, which leads to musculoskeletal system degeneration, deterioration in joint cartilage function, and ultimately, degenerative changes in the spine and limb joints [42]. These degenerative changes increase the risk of sustaining an SCI following falls or other traumatic events [43–45]. Additionally, the prevalence of spinal canal stenosis and ossification of the posterior longitudinal ligament is high among the population [46]. Studies have shown that the relative risk of TSCI with cervical canal stenosis is 124.5 times higher than in patients without it, making spinal injuries more likely to occur from falls [47]. Other contributing factors include a robust industrial and transportation infrastructure leading to a higher incidence of work and vehicular-related TSCI, a vibrant sports culture with activities like judo, rugby, racing, and skiing causing more sports-related TSCI, and a comprehensive healthcare system with high-quality data collection and monitoring that aids in better identification and reporting of TSCI cases. These are the reasons for the higher reported incidence rates of TSCI in Japan. In contrast, the low incidence rate of TSCI in Denmark (10.18 per million) can likely be attributed to its high standards and policies in traffic, occupational, and sports safety, along with strong social welfare system and public health emphasis. This data underscores the critical role of preventive measures in reducing TSCI, such as enhancing safety regulations, raising public awareness, and improving workplace safety. It is noteworthy that our study reports high TSCI incidence rates in Japan and lower rates in Denmark, based on the available research data. In fact, the data for Japan are derived from studies conducted between 2011 and 2018, while the data for Denmark are from studies between 2000 and 2012. Researchers must exercise caution when interpreting these cross-temporal comparisons. This also suggests a need within the current field to further prioritize SCI, by conducting updated epidemiological surveys of SCI to provide the latest information for the prevention and treatment of SCI.

The incidence of SCI increases with age, revealing a complex relationship between aging and the heightened risk of SCI. This correlation is partly due to the increased frequency of falls and other risk factors among the elderly, which are associated with age-related declines in physical function. Additionally, the issue is exacerbated by an aging population, with older individuals are more susceptible to minor injuries that could lead to SCI, such as those caused by osteoporosis. Hence, the healthcare system faces the challenge of addressing the growing need for prevention, diagnosis, and treatment of SCI with age, particularly focusing on the complex rehabilitation and long-term care needs of older patients. Further analysis of the SCI patient population reveals that individuals aged 61 and above constitute 17.54% of SCI cases, which is lower than the proportion of patients aged 21–40 (42.29%) and 41–60 (25.57%). This can be primarily attributed to the larger population base of the 21–40 age group, which could result in a higher number of TSCI cases in this age segment due to the population base effect, even if the relative incidence rate is not the highest. Additionally, the lower proportion of older TSCI patients might also be attributed to the commonality of comorbid conditions in this group, leading to a higher mortality rate.

Gender-based analysis reveals that the incidence of TSCI in males is approximately 3.2 times higher than in females, aligning with the gender ratio of TSCI patients at approximately 3:1, males to females. This significant gender disparity can be attributed to multiple factors, as men are more likely to work in industries with higher occupational hazards, including construction, manufacturing, and transportation. These professions carry a greater risk of falls, heavy machinery accidents, or work-related incidents that could result in SCI [48, 49]. Additionally, socioeconomic and cultural backgrounds may also influence the behavioral patterns of men and women, leading to differences in their susceptibility to SCI. Violence and alcohol abuse significantly contribute to the higher incidence of SCI among men, compared to women. Therefore, the development of prevention strategies and intervention measures must take into account these gender differences to specifically reduce the incidence of TSCI. Moreover, only two studies have found that the risk of injury to the cervical spinal cord is significantly higher than that of the thoracic or lumbar regions [26, 27]. Among patients with TSCI, cervical injuries constitute the largest proportion (47.14%). Although there are no reports comparing the incidence of SCI of varying severities, patients with an AIS grade of A are most common in TSCI cases. These patients, classified with complete injuries, suffer a total loss of sensory and motor functions below the level of injury, leading to the most severe disease burden. This suggests that traumatic events often result in more severe neurological damage. Conversely, a higher proportion of patients with NTSCI have injuries classified as AIS-D, likely related to non-traumatic causes like tumors gradually compressing different neural tissues. We also calculated the proportion of TSCI patients across different occupations. Farmers, workers, and unemployed individuals together account for over 50% of TSCI cases. These groups should be the focus of increased attention in the future. Notably, among the NTSCI population, there are significant differences in the severity of injuries, ages, and injury segments compared to TSCI patients, indicating that NTSCI constitutes a distinct disease entity with significant differences from TSCI. Particularly, the incidence of NTSCI has remained at a high level in recent years, yet epidemiological research related to it is limited. This suggests that future research should pay more attention to NTSCI to enhance awareness for its prevention and treatment.

While this study provides rich information on SCI, the limited quality of evidence included in the research impacts the reliability of the results. This is because 70.74% of the studies originate from hospitals where patient recruitment is passive and not conducted through appropriate sampling surveys, thus limiting the representativeness of the data. Additionally, 43.67% of the studies did not report the diagnostic or measurement standards for SCI, meaning patients with different disease conditions might have been combined for analysis, reducing the reliability of the results. Moreover, 56.33% of the studies did not specify how they addressed confounding factors or conducted subgroup analyses, potentially affecting the accuracy of the reported outcomes and thus decreasing the reliability of the meta-analysis results. Despite conducting several subgroup analyses, there was significant statistical heterogeneity among the included studies. Further, through meta-regression analysis, we found that the quality of the studies, the type of countries, and the types of SCI contributed to the heterogeneity. However, significant heterogeneity persisted after conducting subgroup analyses by country types and injury types, suggesting that the quality of evidence from the included studies may contribute significantly to the heterogeneity. Future research should carefully design trials and strictly adhere to trial implementation and reporting standards to improve the quality of evidence.

## Limitations

The inherent limitations of this study should be acknowledged. Firstly, despite the SRs/MAs covering populations from various regions, the exclusive inclusion of English-language articles might have excluded studies conducted in regions such as Asia, Africa, and Oceania, thereby introducing potential language bias. Secondly, the diagnostic criteria for SCI were not clearly reported in different studies, and there may be inconsistencies in the diagnostic criteria used. Moreover, the majority of the data from the included studies comes from hospitals, excluding individuals who died before hospital arrival or those who returned home due to economic burdens among other reasons. Some studies included data on individuals who died at the scene of the injury, while others overlooked or did not include such cases. Some studies defined motor vehicle accidents as any collision involving a motor vehicle, including those involving pedestrians or cyclists, while others categorized incidents involving pedestrians separately. Finally, to provide a global perspective on the incidence of SCI, we aggregated data on the incidence rates from different times and spaces/countries, which to some extent diminishes the relevance of our findings to the prevention and management of SCI. This also indicates that the current research field requires more up-to-date epidemiological studies to provide the latest evidence for the prevention, diagnosis, and treatment of SCI. In summary, due to the variations in data collection and reporting standards across different studies, the results of the meta-analysis on the incidence and characteristics of SCI patients should be interpreted with caution. However, this research does indeed provide an important data foundation for this under-researched field. Finally, while there is significant heterogeneity among different studies statistically, given that meta-regression analysis attributes this heterogeneity to the quality of studies, country types, and SCI types, the existing heterogeneity is objectively present and must be acknowledged.

## Conclusions

The incidence rates for various types of SCI remain high, with changing demographic characteristics among affected individuals. This study undertook a comprehensive analysis covering SCI categories, data sources, gender, age demographics, temporal and geographic trends, and incidence rates by injury segment. It extensively examined the distribution across gender, injury segments, severity levels, occupational backgrounds, and injury causes, identifying factors contributing to study heterogeneity. Our work aims to serve as a foundation for further research, aiding policymakers, researchers, clinicians, and patients.

### Supplementary Information

Additional file 1: Table S1. [The detailed retrieval strategy of the databases]. Table S2. [Basic information of the included studies]. Table S3. [Annual incidence of TSCI]. Table S4. [Annual incidence of NTSCI]. Table S5. [Incidence in different countries]. Table S6. [Meta-regression analysis results].

## Data Availability

No datasets were generated or analysed during the current study.

## Abbreviations

AIS: American Spinal Injury Association Impairment Scale
NTSCI: Non-traumatic spinal cord injuries
SCI: Spinal cord injury
SRs/MAs: Systematic reviews and meta-analyses
TSCI: Traumatic spinal cord injuries
