# Supplementary material for: Global incidence and characteristics of spinal cord injury since 2000–2021: a systematic review and meta-analysis
Source: BMC Med. 2024 Jul 8;22:285. doi: 10.1186/s12916-024-03514-9 (PMC11229207; doi:10.1186/s12916-024-03514-9)
Supplement: Supplementary file 1 — Additional file 1: Table S1. [The detailed retrieval strategy of the databases]. Table S2. [Basic information of the included studies]. Table S3. [Annual incidence of TSCI]. Table S4. [Annual incidence of NTSCI]. Table S5. [Incidence in different countries]. Table S6. [Meta-regression analysis results]. [file 12916_2024_3514_MOESM1_ESM.docx]

**Global incidence and characteristics of spinal cord injury since 2000-2021: a systematic review and meta-analysis**

**Table S1: The detailed retrieval strategy of the databases**

| **1.Web of Science**  (TS=(Spinal cord injury OR Spinal injury OR Spinal Cord Trauma OR Spinal Cord Transection OR Spinal Cord Laceration OR Post-Traumatic Myelopathy OR Spinal Cord Contusion)) AND TS=(Incidence OR Prevalence OR Epidemiology OR Frequency OR Population OR Survey OR case-control studies OR cohort studies OR hazard OR risk factors OR risk factor OR related factors OR influence factors OR influencing factors OR etiology OR cause OR causes OR pathogeny OR etiological OR reason OR reasons OR causality OR pathogenesis)  **2. PubMed**  #1: "spinal cord injury"[Title/Abstract] OR "spinal injury"[Title/Abstract] OR "spinal cord trauma"[Title/Abstract] OR "spinal cord transection"[Title/Abstract] OR "spinal cord laceration"[Title/Abstract] OR "post traumatic myelopathy"[Title/Abstract] OR "spinal cord contusion"[Title/Abstract]  #2: "Spinal Cord Injuries"[MeSH Terms] OR "Spinal Injuries"[MeSH Terms]  #3: #1 OR #2  #4: (((("Incidence"[Mesh] OR "Epidemiology"[Mesh] OR "Cohort Studies"[Mesh]) OR "Prevalence"[Mesh]) OR "Population"[Mesh]) OR "Surveys and Questionnaires"[Mesh]) OR "Risk Factors"[Mesh] OR "etiology" [Subheading]) OR "Causality"[Mesh])  #5: "Incidence"[Title/Abstract] OR "Prevalence"[Title/Abstract] OR "Epidemiology"[Title/Abstract] OR "Frequency"[Title/Abstract] OR "Population"[Title/Abstract] OR "Survey"[Title/Abstract] OR "case control studies"[Title/Abstract] OR "cohort studies"[Title/Abstract] OR "hazard"[Title/Abstract] OR "risk factors"[Title/Abstract] OR "risk factor"[Title/Abstract] OR "related factors"[Title/Abstract] OR "influence factors"[Title/Abstract] OR "influencing factors"[Title/Abstract] OR "etiology"[Title/Abstract] OR "cause"[Title/Abstract] OR "causes"[Title/Abstract] OR "pathogeny"[Title/Abstract] OR "etiological"[Title/Abstract] OR "reason"[Title/Abstract] OR "reasons"[Title/Abstract] OR "causality"[Title/Abstract] OR "pathogenesis"[Title/Abstract]  #6: #4 OR #5  #7: #3 AND #6  **3.Embase**  #1: 'spinal cord injury':ti,ab,kw OR 'spinal injury':ti,ab,kw OR 'spinal cord trauma':ti,ab,kw OR 'spinal cord transection':ti,ab,kw OR 'spinal cord laceration':ti,ab,kw OR 'post-traumatic myelopathy':ti,ab,kw OR 'spinal cord contusion':ti,ab,kw  #2: 'spinal cord injury'/exp  #3: 'spinal injury'/exp  #4: 'spinal cord trauma'/exp  #5: 'spinal cord transection'/exp  #6: 'spinal cord contusion'/exp  #7: #1 OR #2 OR #3 OR #4 OR #5 OR #6  #8: 'incidence'/exp  #9: 'prevalence'/exp  #10: 'epidemiology'/exp  #11: 'frequency'/exp  #12: 'population'/exp  #13: 'survey'/exp  #14: 'case-control studies'/exp  #15: 'cohort studies'/exp  #16: 'hazard'/exp  #17: 'risk factors'/exp  #18: 'risk factor'/exp  #19: 'pathogenesis'/exp  #20: 'causality'/exp  #21: ' etiology '/exp  #22: incidence:ti,ab,kw OR prevalence:ti,ab,kw OR epidemiology:ti,ab,kw OR frequency:ti,ab,kw OR population:ti,ab,kw OR survey:ti,ab,kw OR 'case-control studies':ti,ab,kw OR 'cohort studies':ti,ab,kw OR hazard:ti,ab,kw OR 'risk factors':ti,ab,kw OR 'risk factor':ti,ab,kw OR 'related factors':ti,ab,kw OR 'influence factors':ti,ab,kw OR 'influencing factors':ti,ab,kw OR etiology:ti,ab,kw OR cause:ti,ab,kw OR causes:ti,ab,kw OR pathogeny:ti,ab,kw OR etiological:ti,ab,kw OR reason:ti,ab,kw OR reasons:ti,ab,kw OR pathogenesis:ti,ab,kw OR causality:ti,ab,kw  #23: #8 OR #9 OR #10 OR #11 OR #12 OR #13 OR #14 OR #15 OR #16 OR #17 OR #18 OR #19 OR #20 OR #21 OR #22  #24: #7 AND #23  **4. Cochrane**  #1: (Spinal cord injury OR Spinal injury OR Spinal Cord Trauma OR Spinal Cord Transection OR Spinal Cord Laceration OR Post-Traumatic Myelopathy OR Spinal Cord Contusion):ti,ab,kw  #2: MeSH descriptor: [Spinal Cord Injuries] explode all trees  #3: MeSH descriptor: [Spinal Injuries] explode all trees  #4: #1 or #2 or #3  #5: (Incidence OR Prevalence OR Epidemiology OR Frequency OR Population OR Survey OR case-control studies OR cohort studies OR hazard OR risk factors OR risk factor OR related factors OR influence factors OR influencing factors OR etiology OR cause OR causes OR pathogeny OR etiological OR reason OR reasons OR causality OR pathogenesis):ti,ab,kw  #6: MeSH descriptor: [Incidence] explode all trees  #7: MeSH descriptor: [Prevalence] explode all trees  #8: MeSH descriptor: [Epidemiology] explode all trees  #9: MeSH descriptor: [Population] explode all trees  #10: MeSH descriptor: [Surveys and Questionnaires] explode all trees  #11: MeSH descriptor: [Case-Control Studies] explode all trees  #12: MeSH descriptor: [Cohort Studies] explode all trees  #13: MeSH descriptor: [Risk Factors] explode all trees  #14: MeSH descriptor: [Causality] explode all trees  #15: #5 or #6 or #7 or #8 or #9 or #10 or #11 or #12 or #13 or #14  #16: #4 and #15  **5.Scopus**  TITLE-ABS-KEY("Spinal cord injury" OR "Spinal injury" OR "Spinal Cord Trauma" OR "Spinal Cord Transection" OR "Spinal Cord Laceration" OR "Post-Traumatic Myelopathy" OR "Spinal Cord Contusion") AND TITLE-ABS-KEY("Incidence" OR "Prevalence" OR "Epidemiology" OR "Frequency" OR "Population" OR "Survey" OR "case-control studies" OR "cohort studies" OR hazard OR "risk factors" OR "risk factor" OR "related factors" OR "influence factors" OR "influencing factors" OR "etiology" OR "cause" OR "causes" OR "pathogeny" OR "etiological" OR "reason" OR "reasons" OR "causality" OR "pathogenesis")  **6.** **ProQuest**  Title(Spinal cord injury OR Spinal injury OR Spinal Cord Trauma OR Spinal Cord Transection OR Spinal Cord Laceration OR Post-Traumatic Myelopathy OR Spinal Cord Contusion)) AND Title(Incidence OR Prevalence OR Epidemiology OR Frequency OR Population OR Survey OR case-control studies OR cohort studies OR hazard OR risk factors OR risk factor OR related factors OR influence factors OR influencing factors OR etiology OR cause OR causes OR pathogeny OR etiological OR reason OR reasons OR causality OR pathogenesis)  **7.** **OpenGrey**  (Title: (Spinal cord injury OR Spinal injury OR Spinal Cord Trauma OR Spinal Cord Transection OR Spinal Cord Laceration OR Post-Traumatic Myelopathy OR Spinal Cord Contusion)) AND Title: (Incidence OR Prevalence OR Epidemiology OR Frequency OR Population OR Survey OR case-control studies OR cohort studies OR hazard OR risk factors OR risk factor OR related factors OR influence factors OR influencing factors OR etiology OR cause OR causes OR pathogeny OR etiological OR reason OR reasons OR causality OR pathogenesis)  **8.** **National Technical Information Service**  (Title: (Spinal cord injury OR Spinal injury OR Spinal Cord Trauma OR Spinal Cord Transection OR Spinal Cord Laceration OR Post-Traumatic Myelopathy OR Spinal Cord Contusion)) AND Title: (Incidence OR Prevalence OR Epidemiology OR Frequency OR Population OR Survey OR case-control studies OR cohort studies OR hazard OR risk factors OR risk factor OR related factors OR influence factors OR influencing factors OR etiology OR cause OR causes OR pathogeny OR etiological OR reason OR reasons OR causality OR pathogenesis)  **9.** **WHO International Clinical Trials Registry Platform**  Condition (Spinal cord injury) AND Title (Incidence OR Prevalence OR Epidemiology OR Frequency OR Population OR Survey OR case-control studies OR cohort studies OR hazard OR risk factors OR risk factor OR related factors OR influence factors OR influencing factors OR etiology OR cause OR causes OR pathogeny OR etiological OR reason OR reasons OR causality OR pathogenesis)  **10** **the US National Institutes of Health**  Condition (Spinal cord injury) AND Title (Incidence OR Prevalence OR Epidemiology OR Frequency OR Population OR Survey OR case-control studies OR cohort studies OR hazard OR risk factors OR risk factor OR related factors OR influence factors OR influencing factors OR etiology OR cause OR causes OR pathogeny OR etiological OR reason OR reasons OR causality OR pathogenesis) |
| --- |

**Table S2: Basic information of the included studies**

| **NO.** | **First author** | **Year** | **Study type** | **Research time** | **Country** | **Source of patient** | **Sample size** |
| --- | --- | --- | --- | --- | --- | --- | --- |
| 1 | Meng | 2023 | Retrospective study | 2011-2020 | China | Hospital | 86 |
| 2 | Miyakoshi | 2020 | Retrospective study | 2018 | Japan | Hospital | 4603 |
| 3 | Berg | 2017 | Cohort study | 2001-2008 | Spain | Hospital | 58 |
| 4 | Moshi | 2021 | Cohort study | 2017 | Tanzania | Hospital | 87 |
| 5 | Bakhsh | 2020 | Cohort study | 2017.01-2018.06 | Kingdom of Saudi Arabia | Hospital | 230 |
| 6 | Hua | 2013 | Retrospective study | 2001-2010 | China | Hospital | 561 |
| 7 | Shin | 2013 | Retrospective study | 2004-2008 | South Korea | Hospital | 629 |
| 8 | Chen | 2013 | Retrospective study | 2005-2011 | USA | Database | 7834 |
| 9 | Koskinen | 2014 | Cohort study | 2012 | Finland | Database | 77 |
| 10 | McCaughey | 2016 | Cohort study | 2004-2013 | England | Database | 1235 |
| 11 | Islam | 2011 | Cross sectional study | 2009 | Bangladesh | Hospital | 107 |
| 12 | Yusuf | 2019 | Retrospective study | 2014-2016 | Nigeria | Hospital | 133 |
| 13 | Middleton | 2023 | Cross sectional study | 2018-2019 | Australia | Community | 1579 |
| 14 | Bellucci | 2013 | Cross sectional study | 2012 | Brazil | Hospital | 348 |
| 15 | Wu | 2022 | Retrospective study | 2010-2019 | China | Hospital | 649 |
| 16 | Gatti | 2020 | Cross sectional study | 2015-2019 | Argentina | Hospital | 186 |
| 17 | Zárate | 2016 | Retrospective study | 2005-2012 | Mexico | Hospital | 433 |
| 18 | Korkmaz | 2021 | Retrospective study | 2016-2020 | Turkey | Hospital | 484 |
| 19 | Lee | 2021 | Retrospective study | 2000-2019 | South Korea | Hospital | 861 |
| 20 | TUĞCU | 2011 | Retrospective study | 2000-2007 | Turkey | Hospital | 905 |
| 21 | Lee | 2021 | Retrospective study | 2000-2019 | South Korea | Hospital | 2707 |
| 22 | Jakimovska | 2019 | Cohort study | 2015-2016 | Republic of Macedonia | Hospital | 38 |
| 23 | Guo | 2012 | Retrospective study | 2004-2008 | China | Hospital | 203 |
| 24 | Sun | 2021 | Retrospective study | 2009-2018 | China | Hospital | 503 |
| 25 | Wang | 2022 | Cohort study | 2017-2020 | China | Hospital | 2621 |
| 26 | Du | 2020 | Cohort study | 2014-2018 | China | Hospital | 1730 |
| 27 | BRITO | 2011 | Cross sectional study | 2008-2009 | Brazil | Hospital | 87 |
| 28 | Liu | 2020 | Retrospective study | 2011-2019 | China | Hospital | 590 |
| 29 | Ning | 2015 | Retrospective study | 2009-2013 | China | Hospital | 554 |
| 30 | Chen | 2020 | Retrospective study | 2011-2015 | China | Hospital | 482 |
| 31 | Faleiros | 2023 | Cross sectional study | 2018 | Brazil | Community | 618 |
| 32 | Shibahashi | 2018 | Cohort study | 2004-2015 | Japan | Database | 8069 |
| 33 | Chamberlain | 2015 | Cohort study | 2005-2012 | Switzerland | Hospital | 932 |
| 34 | Sothmann | 2015 | Retrospective study | 2003-2014 | South Africa | Database | 2042 |
| 35 | Smith | 2020 | Cohort study | 2017 | Ireland | Hospital | 129 |
| 36 | Musubire | 2019 | Cohort study | 2013-2015 | Uganda | Hospital | 103 |
| 37 | Smith | 2017 | Retrospective study | 2000-2015 | Ireland | Database | 48 |
| 38 | Saunders | 2015 | Cohort study | 2000-2012 | USA | Database | 376 |
| 39 | Æsøy | 2015 | Retrospective study | 2004-2013 | Norway | Hospital | 23 |
| 40 | Yang | 2014 | Retrospective study | 2003-2011 | China | Hospital | 3832 |
| 41 | Mehdar | 2019 | Retrospective study | 2018-2019 | Saudi Arabia | Hospital | 182 |
| 42 | Ibrahim | 2013 | Cross sectional study | 2006-2009 | Malaysia | Hospital | 292 |
| 43 | Darain | 2016 | Retrospective study | 2011-2016 | Pakistan | Hospital | 1025 |
| 44 | Knox | 2016 | Retrospective study | 2012 | USA | Database | 297 |
| 45 | Kim | 2021 | Cross sectional study | 2012-2018 | South Korea | Hospital | 221 |
| 46 | Kristinsdóttir | 2018 | Retrospective study | 2007-2011 | Iceland | Hospital | 42 |
| 47 | Piatt | 2018 | Retrospective study | 2000-2012 | USA | Database | / |
| 48 | Wu | 2012 | Retrospective study | 2008-2011 | China | Hospital | 143 |
| 49 | Wang | 2013 | Cross sectional study | 2001-2010 | China | Hospital | 417 |
| 50 | Majdan | 2015 | Retrospective study | 2002–2012 | Austria | Database | 1543 |
| 51 | Giraldo | 2021 | Cohort study | 2009-2012 | Colombia | Hospital | 258 |
| 52 | Knutsdottir | 2012 | Retrospective study | 2000-2009 | Iceland | Hospital | 82 |
| 53 | Nijendijk | 2014 | Retrospective study | 2010 | Netherlands | Database | 185 |
| 54 | Johansson | 2020 | Cohort study | 2012-2015 | Finland | Hospital | 271 |
| 55 | Montoto | 2017 | Retrospective study | 2000-2014 | Spain | Hospital | 860 |
| 56 | Bárbara | 2017 | Retrospective study | 2000-2014 | Spain | Hospital | 141 |
| 57 | Smith | 2018 | Retrospective study | 2010–2015 | Ireland | Database | 347 |
| 58 | Halvorsen | 2018 | Cross sectional study | 2012-2016 | Norway | Hospital | 349 |
| 59 | Ning | 2011 | Retrospective study | 2004-2008 | China | Hospital | 869 |
| 60 | Li | 2018 | Retrospective study | 2002-2016 | China | Hospital | 680 |
| 61 | Amidei | 2022 | Retrospective study | 2011-2020 | Italy | Database | 1303 |
| 62 | Jonviea | 2017 | Cohort study | 2012-2013 | Switzerland | Database | 621 |
| 63 | Ge | 2019 | Retrospective study | 2003-2014 | USA | Hospital | 30 |
| 64 | Ullah | 2023 | Cohort study | 2014 | Pakistan | Hospital | 384 |
| 65 | Chen | 2021 | Retrospective study | 2015-2019 | USA | Database | 3606 |
| 66 | Katoh | 2014 | Retrospective study | 2011-2012 | Japan | Hospital | 185 |
| 67 | Sabre | 2012 | Cohort study | 2000-2007 | Estonia | Hospital | 595 |
| 68 | SHARIF | 2014 | Retrospective study | 2010-2011 | Iran | Hospital | 867 |
| 69 | Rahimi | 2008 | Cross sectional study | 2003-2008 | Iran | Community | / |
| 70 | GHAJARZADEH | 2019 | Cohort study | 2013-2017 | Iran | Hospital | 830 |
| 71 | Sabre | 2014 | Retrospective study | 2005-2007 | Estonia | Hospital | 391 |
| 72 | Joseph | 2015 | Cohort study | 2013-2014 | South Africa | Hospital | 147 |
| 73 | Schoenfeld | 2011 | Cohort study | 2000–2009 | USA | Database | 5928 |
| 74 | Li | 2023 | Retrospective study | 2013-2019 | China | Hospital | 164 |
| 75 | Wang | 2016 | Cross sectional study | 2001-2011 | China | Hospital | 698 |
| 76 | Noonan | 2012 | Cross sectional study | 2010 | Canada | Database | / |
| 77 | Niemi | 2020 | Cohort study | 2013-2016 | Finland | Hospital | 430 |
| 78 | Kriz | 2017 | Cohort study | 2006-2015 | Czech Republic | Hospital | 2642 |
| 79 | Mirzaeva | 2019 | Cohort study | 2012-2016 | Russia | Database | 361 |
| 80 | Rau | 2022 | Retrospective study | 2013-2020 | Germany | Database | 10360 |
| 81 | Berg | 2011 | Cohort study | 2001-2008 | Spain | Hospital | 133 |
| 82 | Noe | 2015 | Cohort study | 2000-2012 | Denmark | Hospital | 409 |
| 83 | Ferro | 2017 | Cohort study | 2013-2014 | Italy | Hospital | 445 |
| 84 | Pérez | 2012 | Retrospective study | 2000–2009 | Spain | Database | 3698 |
| 85 | Joseph | 2017 | Cohort study | 2014-2015 | Sweden | Database | 45 |
| 86 | Selvarajah | 2015 | Retrospective study | 2006-2011 | USA | Database | 117444 |
| 87 | Divanoglou | 2009 | Cohort study | 2006 | Greece, Sweden | Hospital | 128 |
| 88 | ERDOĞAN | 2013 | Retrospective study | 2007-2011 | Turkey | Hospital | 409 |
| 89 | Andalib | 2018 | Cross sectional study | 2015-2018 | Iran | Hospital | 127 |
| 90 | Wilson | 2020 | Cohort study | 2002-2017 | Canada | Database | 1865 |
| 91 | Halvorsen | 2018 | Cross sectional study | 2012–2016 | Norway | Database | 225 |
| 92 | Gupta | 2009 | Cross sectional study | 2005-2008 | India | Hospital | 64 |
| 93 | Kanna | 2021 | Retrospective study | 2015-2019 | India | Hospital | 2065 |
| 94 | Augutis | 2006 | Cross sectional study | 2002-2004 | 19 countries | Hospital | / |
| 95 | Joseph | 2015 | Cross sectional study | 2009 | USA | Database | / |
| 96 | James | 2022 | Cross sectional study | 2016 | USA | Database | 1281 |
| 97 | Avila | 2020 | Retrospective study | 2006-2015 | USA | Database | 20309 |
| 98 | Moorin | 2014 | Cohort study | 2002-2008 | Australia | Database | 335 |
| 99 | Jiang | 2022 | Cross sectional study | 2013 | China | Community | / |
| 100 | Dumura | 2023 | Cohort study | 2001-2011 | Nigeria | Hospital | 296 |
| 101 | Smith | 2019 | Cohort study | 2016 | Ireland | Database | 61 |
| 102 | Vervoordeldonk | 2013 | Cohort study | 2006-2010 | Netherlands | Hospital | 124 |
| 103 | Albert | 2005 | Cohort study | 2000 | France | Hospital | / |
| 104 | Smith | 2014 | Retrospective study | 2001-2010 | Ireland | Database | / |
| 105 | Cruz | 2015 | Retrospective study | 2006-2013 | Spain | Hospital | 48 |
| 106 | Júnior | 2011 | Retrospective study | 2005-2008 | Brazil | Hospital | 54 |
| 107 | MORAIS | 2012 | Cross sectional study | 2008-2012 | Brazil | Hospital | 321 |
| 108 | Derakhshanrad | 2016 | Cross sectional study | 2011-2015 | Iran | Hospital | 1137 |
| 109 | Khazaeipour | 2017 | Cross sectional study | 2012-2013 | Iran | Hospital | 140 |
| 110 | Lieutaud | 2012 | Retrospective study | 2003-2008 | France | Hospital | 82 |
| 111 | Noreau | 2014 | Cross sectional study | 2011-2012 | Canada | Community | 1549 |
| 112 | McCammon | 2011 | Cohort study | 2003-2007 | Canada | Database | 250 |
| 113 | Guzelkucuk | 2014 | Retrospective study | 2010-2013 | Turkey | Hospital | 148 |
| 114 | Mathur | 2014 | Cohort study | 2000-2008 | India | Hospital | 2716 |
| 115 | Spota | 2023 | Retrospective study | 2010-2020 | Italy | Database | 180 |
| 116 | Lien | 2021 | Retrospective study | 2002-2015 | China | Database | 5048 |
| 117 | Selvarajah | 2014 | Retrospective study | 2007-2009 | USA | Database | 43137 |
| 118 | Liu | 2020 | Retrospective study | 2013-2019 | China | Hospital | 2448 |
| 119 | Thompson | 2014 | Cohort study | 2000-2010 | Canada | Hospital | 831 |
| 120 | Willott | 2020 | Cohort study | 2015-2016 | Nepal | Hospital | 184 |
| 121 | Li | 2011 | Cross sectional study | 2002 | China | Hospital | 264 |
| 122 | Selvarajah | 2014 | Retrospective study | 2007-2010 | USA | Database | 6132 |
| 123 | Oteir | 2016 | Cohort study | 2007-2012 | Australia | Database | 106059 |
| 124 | Lenehan | 2012 | Retrospective study | 2000-2004 | Canada | Database | / |
| 125 | Vedantam | 2022 | Retrospective study | 2005-2019 | North America | Database | 746 |
| 126 | Middleton | 2012 | Retrospective study | 2004-2008 | Australia | Database | 324 |
| 127 | Tsolinas | 2020 | Cohort study | 2015-2019 | USA | Database | 160 |
| 128 | Derrett | 2012 | Cross sectional study | 2007-2009 | New Zealand | Hospital | 118 |
| 129 | Tholl | 2023 | Cross sectional study | 2019-2020 | Brazil | Hospital | 91 |
| 130 | Zhang | 2021 | Retrospective study | 2010-2019 | China | Hospital | 1858 |
| 131 | Löfvenmark | 2015 | Cross sectional study | 2011-2013 | Sweden | Hospital | 49 |
| 132 | Alshahri | 2012 | Retrospective study | 2003-2008 | Saudi Arabia | Hospital | 307 |
| 133 | Moshi | 2017 | Retrospective study | 2010-2014 | Tanzania | Hospital | 213 |
| 134 | Zhou | 2015 | Retrospective study | 2009-2014 | China | Hospital | 354 |
| 135 | Parajuli | 2023 | Cross sectional study | 2019-2021 | Nepal | Hospital | 164 |
| 136 | Aarabi | 2021 | Retrospective study | 2001-2018 | USA | Hospital | 1420 |
| 137 | Gwynedd | 2006 | Retrospective study | 2000 | Canada | Hospital | / |
| 138 | Sabre | 2022 | Cohort study | 2000-2018 | Norway | Hospital | 383 |
| 139 | Hao | 2021 | Retrospective study | 2009-2018 | China | Database | / |
| 140 | Steinemann | 2019 | Retrospective study | 2009-2017 | USA | Database | 942 |
| 141 | Franceschini | 2020 | Cohort study | 2013-2014 | Italy | Hospital | 497 |
| 142 | Rahman | 2017 | Retrospective study | 2011-2016 | Bangladesh | Hospital | 2184 |
| 143 | Wang | 2020 | Retrospective study | 2014-2018 | China | Hospital | 3487 |
| 144 | Bárbara | 2018 | Cohort study | 2001-2015 | Spain | Hospital | 282 |
| 145 | O’Connor | 2006 | Cohort study | 2000 | Ireland | Hospital | 46 |
| 146 | Agarwal | 2007 | Cross sectional study | 2003-2004 | India | Hospital | 207 |
| 147 | New | 2008 | Retrospective study | 2000-2006 | Australia | Database | / |
| 148 | Taşoğlu | 2016 | Retrospective study | 2013-2014 | Turkey | Hospital | 262 |
| 149 | Chang | 2017 | Cross sectional study | 2000-2015 | China | Community | 514 |
| 150 | Wu | 2013 | Retrospective study | 2004-2007 | China | Hospital | 631 |
| 151 | Eaton | 2018 | Cohort study | 2016-2017 | Malawi | Hospital | 46 |
| 152 | Ones | 2007 | Retrospective study | 2002-2005 | Turkey | Hospital | 194 |
| 153 | Yang | 2017 | Retrospective study | 2003-2011 | China | Hospital | 1336 |
| 154 | Wu | 2022 | Retrospective study | 2012-2018 | China | Hospital | 1290 |
| 155 | Shrestha | 2013 | Retrospective study | 2008-2011 | Nepal | Hospital | 381 |
| 156 | DeVivo | 2011 | Cohort study | 2000-2008 | USA | Hospital | 9583 |
| 157 | Maayken | 2012 | Retrospective study | 2001-2008 | Spain | Hospital | 161 |
| 158 | Chen | 2016 | Cross sectional study | 2000-2014 | USA | Database | 10610 |
| 159 | Jørgensen | 2016 | Cross sectional study | 2013-2014 | Sweden | Hospital | 224 |
| 160 | Ge | 2017 | Retrospective study | 2003-2013 | USA | Hospital | 685 |
| 161 | Jain | 2015 | Retrospective study | 2012 | USA | Database | / |
| 162 | Yang | 2011 | Retrospective study | 2006-2009 | South Korea | Hospital | 47 |
| 163 | Joseph | 2023 | Cross sectional study | 2017-2018 | South Africa | Community | 200 |
| 164 | Shang | 2023 | Retrospective study | 2013-2018 | China | Hospital | 13465 |
| 165 | Toda | 2018 | Retrospective study | 2004-2013 | Japan | Hospital | 279 |
| 166 | Arul | 2019 | Retrospective study | 2003-2013 | USA | Hospital | 147 |
| 167 | Beck | 2019 | Cohort study | 2007-2016 | Australia | Database | 706 |
| 168 | New | 2014 | Cross sectional study | 2011 | Australia | Hospital | / |
| 169 | Chaurasia | 2006 | Cohort study | 2002-2004 | India | Hospital | 126 |
| 170 | Bellet | 2019 | Retrospective study | 2012-2016 | Tanzania | Hospital | 105 |
| 171 | Kudo | 2019 | Retrospective study | 2012-2016 | Japan | Hospital | 449 |
| 172 | Choi | 2019 | Cohort study | 2007-2017 | South Korea | Database | / |
| 173 | Feng | 2021 | Retrospective study | 2013-2017 | China | Hospital | 338 |
| 174 | Prasad | 2017 | Retrospective study | 2010-2015 | Kuwait | Hospital | 241 |
| 175 | Barbetta | 2018 | Cross sectional study | 2014 | Brazil | Database | 1505 |
| 176 | Phillips | 2018 | Cohort study | 2013-2014 | South Africa | Hospital | 158 |
| 177 | Costacurta | 2010 | Retrospective study | 2002-2008 | Brazil | Hospital | 106 |
| 178 | New | 2011 | Cohort study | 2002-2006 | Australia | Hospital | 3610 |
| 179 | Chhabra | 2012 | Retrospective study | 2002-2010 | India | Hospital | 1138 |
| 180 | Wang | 2013 | Retrospective study | 2007-2010 | China | Hospital | 761 |
| 181 | Equebal | 2013 | Retrospective study | 2009-2010 | India | Hospital | 47 |
| 182 | Mahabaleshwarkar | 2014 | Retrospective study | 2009 | USA | Database | 11848 |
| 183 | Güzelküçük | 2014 | Retrospective study | 2009-2013 | Turkey | Hospital | 242 |
| 184 | Rodríguez | 2015 | Cross sectional study | 2006-2013 | Mexico | Hospital | 464 |
| 185 | Couris | 2010 | Cohort study | 2003-2006 | Canada | Database | 936 |
| 186 | Nwankwo | 2013 | Retrospective study | 2009-2012 | Nigeria | Hospital | 85 |
| 187 | Güzelküçük | 2016 | Retrospective study | 2007-2013 | Turkey | Hospital | 102 |
| 188 | Ametefe | 2016 | Retrospective study | 2012-2014 | Ghana | Hospital | 185 |
| 189 | Chen | 2017 | Retrospective study | 2009-2013 | China | Hospital | 232 |
| 190 | Sebastia | 2013 | Retrospective study | 2001-2013 | Spain | Hospital | / |
| 191 | Selassie | 2015 | Retrospective study | 2012 | USA | Database | / |
| 192 | Feng | 2011 | Retrospective study | 2000-2009 | China | Hospital | 191 |
| 193 | Chen | 2015 | Cohort study | 2005-2014 | USA | Hospital | 5868 |
| 194 | Rashid | 2017 | Retrospective study | 2011-2015 | Tanzania | Hospital | 125 |
| 195 | Deconinck | 2003 | Cross sectional study | 2001 | Switzerland | Community | 311 |
| 196 | Rathore | 2007 | Cross sectional study | 2005 | Pakistan | Hospital | 187 |
| 197 | Singh | 2019 | Cross sectional study | 2013-2014 | India | Hospital | 157 |
| 198 | Singh | 2003 | Cohort study | 2000-2001 | India | Hospital | 483 |
| 199 | Crul | 2023 | Cohort study | 2013-2019 | Netherlands | Hospital | 1432 |
| 200 | Furlan | 2023 | Cohort study | 2004-2019 | Canada | Hospital | 5571 |
| 201 | Raguindin | 2023 | Cohort study | 2013-2021 | Switzerland | Hospital | 706 |
| 202 | Khadour | 2023 | Retrospective study | 2016-2021 | China | Hospital | 463 |
| 203 | Conti | 2023 | Cohort study | 2008-2020 | Italy | Hospital | 892 |
| 204 | Gidu | 2023 | Retrospective study | 2017-2021 | Romania | Database | 96 |
| 205 | Bae | 2023 | Retrospective study | 2010-2019 | South Korea | Database | 3855 |
| 206 | Wang | 2023 | Retrospective study | 2012-2020 | China | Hospital | 1806 |
| 207 | Choi | 2023 | Retrospective study | 2009-2018 | South Korea | Database | / |
| 208 | Choi | 2023 | Retrospective study | 2007-2020 | South Korea | Database | / |
| 209 | Zou | 2023 | Retrospective study | 2007-2019 | China | Hospital | 187 |
| 210 | Paiva | 2023 | Retrospective study | 2019 | Brazil | Hospital | 41 |
| 211 | Wilson | 2022 | Retrospective study | 2016-2020 | Ireland | Hospital | 42 |
| 212 | Alves | 2023 | Retrospective study | 2018-2019 | South Africa | Hospital | 386 |
| 213 | Mugisa | 2023 | Cross sectional study | 2019 | Uganda | Hospital | 103 |
| 214 | Yang | 2023 | Retrospective study | 2016-2020 | Korea | Hospital | 363 |
| 215 | Neyaz | 2024 | Cross sectional study | 2018-2021 | India | Hospital | 167 |
| 216 | Saleh | 2023 | Cross sectional study | 2022 | Lebanon | Hospital | 81 |
| 217 | Lau | 2014 | Retrospective study | 2000-2009 | Australia | Hospital | 206 |
| 218 | Thorogood | 2023 | Retrospective study | 2005-2016 | Canada | Database | 1199 |
| 219 | Lee | 2024 | Retrospective study | 2014-2020 | USA | Database | 2219 |
| 220 | Crispo | 2022 | Cross sectional study | 2016 | USA | Database | 1281 |
| 221 | Gautam | 2023 | Cross sectional study | 2022 | Nepal | Hospital | 316 |
| 222 | Kennedy | 2016 | Retrospective study | 2008-2015 | England | Hospital | 265 |
| 223 | Niemeyer | 2020 | Retrospective study | 2015-2018 | Netherlands | Hospital | 151 |
| 224 | Altahla | 2023 | Retrospective study | 2019-2023 | China | Hospital | 93 |
| 225 | Uddin | 2023 | Cross sectional study | 2018-2022 | Bangladesh | Hospital | 3035 |
| 226 | Zhou | 2024 | Retrospective study | 2013-2018 | China | Hospital | 13465 |
| 227 | Mahmoud | 2017 | Cohort study | 2009-2014 | Saudi Arabia | Hospital | 418 |
| 228 | Lee | 2023 | Retrospective study | 2014-2020 | USA | Database | 2807 |
| 229 | Tong | 2024 | Retrospective study | 2011-2020 | China | Hospital | 351 |

**Table S3: Annual incidence of TSCI**

| **Year** | **Effect size** | **Lower limit of 95% CI** | **Upper limit of 95% CI** | **Number of studies** |
| --- | --- | --- | --- | --- |
| 2000 | 24.89 | 16.60 | 34.86 | 9 |
| 2001 | 25.51 | 18.35 | 33.86 | 6 |
| 2002 | 26.99 | 17.95 | 37.86 | 9 |
| 2003 | 23.33 | 17.13 | 30.48 | 8 |
| 2004 | 27.33 | 19.98 | 35.83 | 8 |
| 2005 | 26.13 | 19.87 | 33.25 | 8 |
| 2006 | 24.11 | 17.72 | 31.49 | 10 |
| 2007 | 30.43 | 20.56 | 42.23 | 8 |
| 2008 | 23.73 | 16.03 | 32.93 | 8 |
| 2009 | 26.09 | 18.43 | 35.08 | 10 |
| 2010 | 20.87 | 15.01 | 27.68 | 12 |
| 2011 | 25.28 | 11.83 | 43.79 | 9 |
| 2012 | 31.20 | 18.05 | 47.93 | 14 |
| 2013 | 25.01 | 16.13 | 35.83 | 9 |
| 2014 | 22.47 | 13.50 | 33.70 | 7 |
| 2015 | 23.56 | 14.41 | 34.95 | 7 |
| 2016 | 22.66 | 12.98 | 35.02 | 7 |
| 2017 | 35.07 | 16.29 | 60.97 | 7 |
| 2018 | 44.53 | 22.89 | 73.31 | 8 |
| 2019 | 41.62 | 5.80 | 110.11 | 4 |
| 2020 | 40.96 | 1.23 | 136.68 | 3 |
| 2021 | 32.40 | 31.29 | 33.54 | 1 |

**Table S4: Annual incidence of NTSCI**

| **Year** | **Effect size** | **Lower limit of 95% CI** | **Upper limit of 95% CI** | **Number of studies** |
| --- | --- | --- | --- | --- |
| 2000 | 3.80 | 3.43 | 4.20 | 2 |
| 2001 | 2.20 | 1.92 | 2.51 | 1 |
| 2002 | 3.20 | 2.86 | 3.57 | 1 |
| 2003 | 3.00 | 2.67 | 3.36 | 1 |
| 2004 | 1.00 | 0.81 | 1.22 | 1 |
| 2005 | 1.80 | 1.55 | 2.08 | 1 |
| 2006 | 2.90 | 2.58 | 3.25 | 1 |
| 2007 | 11.41 | 10.95 | 11.88 | 2 |
| 2008 | 12.96 | 2.39 | 31.96 | 3 |
| 2009 | 10.78 | 1.04 | 30.74 | 3 |
| 2010 | 12.27 | 1.74 | 32.32 | 3 |
| 2011 | 12.37 | 0.80 | 37.66 | 3 |
| 2012 | 16.62 | 6.83 | 30.68 | 6 |
| 2013 | 19.51 | 6.39 | 39.78 | 6 |
| 2014 | 29.61 | 10.07 | 59.44 | 5 |
| 2015 | 34.93 | 12.81 | 67.91 | 5 |
| 2016 | 41.89 | 12.35 | 88.92 | 5 |
| 2017 | 25.97 | 3.36 | 69.87 | 3 |
| 2018 | 32.16 | 31.38 | 32.96 | 2 |
| 2019 | 30.56 | 29.80 | 31.33 | 2 |
| 2020 | 26.73 | 26.02 | 27.45 | 2 |

**Table S5: Incidence in different countries**

| **Country** | **Effect size** | **Lower limit of 95% CI** | **Upper limit of 95% CI** |
| --- | --- | --- | --- |
| Japan | 95.25 | 62.80 | 134.43 |
| Tanzania | 31.72 | 30.94 | 32.50 |
| Finland | 30.58 | 29.82 | 31.35 |
| England | 17.06 | 16.38 | 17.74 |
| Former Yugoslav Republic of Macedonia | 13.00 | 12.30 | 13.73 |
| China | 63.34 | 45.37 | 84.29 |
| Switzerland | 40.26 | 24.64 | 59.70 |
| USA | 30.88 | 24.11 | 38.49 |
| Iceland | 23.91 | 23.23 | 24.59 |
| Austria | 16.85 | 15.50 | 18.26 |
| Colombia | 44.15 | 43.24 | 45.08 |
| Netherlands | 14.00 | 13.28 | 14.75 |
| Spain | 14.00 | 10.25 | 18.34 |
| Ireland | 12.95 | 12.47 | 13.44 |
| Norway | 27.06 | 22.99 | 31.48 |
| Estonia | 44.31 | 34.87 | 54.88 |
| Iran | 24.37 | 23.69 | 25.06 |
| South Africa | 68.16 | 67.02 | 69.31 |
| Canada | 24.07 | 20.65 | 27.76 |
| Czech Republic | 16.50 | 15.71 | 17.32 |
| Russia | 16.62 | 14.12 | 19.32 |
| Denmark | 10.18 | 8.24 | 12.32 |
| Italy | 10.21 | 8.98 | 11.52 |
| Sweden | 15.86 | 15.31 | 16.42 |
| Australia | 21.40 | 11.45 | 34.44 |
| France | 10.37 | 9.93 | 10.82 |
| New Zealand | 30.00 | 28.94 | 31.09 |
| South Korea | 40.46 | 35.24 | 46.04 |
| Romania | 24.33 | 18.49 | 30.97 |

**Table S6: Meta-regression analysis results**

| **Year** | | | | | | |
| --- | --- | --- | --- | --- | --- | --- |
| **ES** | **exp(b)** | **Std. Err.** | **t** | **P>\|t\|** | **[95% Conf. Interval]** | |
| year | 1.012455 | 0.0117102 | 1.07 | 0.285 | 0.9896739 | 1 03576 |
| _cons | 2.86e-16 | 6.67e-15 | -1.53 | 0.126 | 3.29e-36 | 24824.2 |
| **Quality of Study** | | | | | | |
| **ES** | **exp(b)** | **Std. Err.** | **t** | **P>\|t\|** | **[95% Conf. Interval]** | |
| quality1 | 1.357822 | 0.1558131 | 2.67 | 0.008 | 1.083385 | 1.701779 |
| quality2 | 2.079837 | 0.3147008 | 4.84 | 0.000 | 1.54429 | 2.801107 |
| _cons | 0.0000147 | 1.44e-06 | -113.27 | 0.000 | 0.0000121 | 0.0000178 |
| **Data Source** | | | | | | |
| **ES** | **exp(b)** | **Std. Err.** | **t** | **P>\|t\|** | **[95% Conf. Interval]** | |
| datasource1 | 2.374525 | 1.436132 | 1.43 | 0.154 | 0.7223464 | 7.80563 |
| datasource2 | 1.01623 | 0.0980595 | 0.17 | 0.868 | 0.840494 | 1.228709 |
| _cons | 0.0000197 | 1.38e-06 | -154.62 | 0.000 | 0.0000172 | 0.0000226 |
| **Country Type** | | | | | | |
| **ES** | **exp(b)** | **Std. Err.** | **t** | **P>\|t\|** | **[95% Conf. Interval]** | |
| countrytype1 | 0.552506 | 0.0801894 | -4.09 | 0.000 | 0.4152533 | 0.7351246 |
| _cons | 0.0000337 | 4.600-06 | -75.55 | 0.000 | 0.0000258 | 0. 0000441 |
| **Study Type** | | | | | | |
| **ES** | **exp(b)** | **Std. Err.** | **t** | **P>\|t\|** | **[95% Conf. Interval]** | |
| studytype1 | 0.8747304 | 0.1670863 | -0.70 | 0.484 | 0.6006825 | 1.273806 |
| studytype3 | 1.137781 | 0.2223179 | 0.66 | 0.509 | 0.774614 | 1.671214 |
| _cons | 0.0000205 | 3.68e-06 | -60.07 | 0.000 | 0.0000144 | 0.0000291 |
| **SCI Type** | | | | | | |
| **ES** | **exp(b)** | **Std. Err.** | **t** | **P>\|t\|** | **[95% Conf. Interval]** | |
| scitype1 | 0.096034 | 0.0764024 | -2.95 | 0.003 | 0.0200705 | 0.4595071 |
| scitype2 | 0.4873259 | 0.0567128 | -6.18 | 0.000 | 0.3875874 | 0.6127302 |
| scitype3 | 0.7230755 | 0.1085912 | -2.16 | 0.032 | 0.5380792 | 0.971675 |
| scitype4 | 0.2396674 | 0.1906737 | -1.80 | 0.074 | 0.0500889 | 1.14677 |
| _cons | 0.0000239 | 1.28e-06 | -198.81 | 0.000 | 0.0000216 | 0.0000266 |
| **Country** | | | | | | |
| **ES** | **exp(b)** | **Std. Err.** | **t** | **P>\|t\|** | **[95% Conf. Interval]** | |
| country1 | 0.7296989 | 0.4793668 | -0.48 | 0.632 | 0.2002382 | 2.659135 |
| country2 | 0.559237 | 0.3420159 | -0.95 | 0.343 | 0.1677945 | 1.863863 |
| country3 | 0.7643576 | 0.4573651 | -0.45 | 0.654 | 0.2353812 | 2.482112 |
| country4 | 1.687784 | 1.022235 | 0.86 | 0.388 | 0.5123304 | 5.56011 |
| country5 | 1.447237 | 1.041489 | 0.51 | 0.608 | 0.3510275 | 5.966752 |
| country6 | 0.5090544 | 0.3453845 | -1.00 | 0.321 | 0.1338904 | 1.935436 |
| country7 | 0.337521 | 0.2290021 | -1.60 | 0.111 | 0.0887741 | 1.283262 |
| country8 | 0.2333448 | 0.1393759 | -2.44 | 0.015 | 0.0720091 | 0.7561513 |
| country9 | 1.440084 | 0.8874693 | 0.59 | 0.554 | 0.4281178 | 4.84409 |
| country10 | 1.53085 | 0.9540657 | 0.68 | 0.495 | 0.4489058 | 5.220477 |
| country11 | 0.4333336 | 0.3600863 | -1.01 | 0.315 | 0.0844214 | 2.224295 |
| country12 | 0.3415 | 0.2457569 | -1.49 | 0.137 | 0.0828309 | 1.407956 |
| country13 | 0.5243332 | 0.4357041 | -0.78 | 0.438 | 0.1021498 | 2.691394 |
| country14 | 1.12 | 0.9306844 | 0.14 | 0.892 | 0.2181967 | 5.748944 |
| country15 | 0.7937255 | 0.5711961 | -0.32 | 0.748 | 0.1925183 | 3.272418 |
| country16 | 0.7164731 | 0.5156023 | -0.46 | 0.644 | 0.1737807 | 2.953917 |
| country17 | 0.4562884 | 0.2782282 | -1.29 | 0.199 | 0.1373948 | 1.515334 |
| country18 | 0.2915789 | 0.1742557 | -2.06 | 0.040 | 0.0899211 | 0.945476 |
| country19 | 3.075248 | 2.020247 | 1.71 | 0.088 | 0.8438851 | 11.20668 |
| country20 | 1.416721 | 0.848299 | 0.58 | 0.561 | 0.4359216 | 4.604267 |
| country21 | 0.4656666 | 0.387785 | -0.92 | 0.360 | 0.0909153 | 2.395392 |
| country22 | 0.6722078 | 0.401298 | -0.67 | 0.506 | 0.2075672 | 2.17695 |
| country23 | 0.7907307 | 0.501847 | -0.37 | 0.712 | 0.2267121 | 2.757926 |
| country24 | 0.5493992 | 0.3486829 | -0.94 | 0.346 | 0.1575194 | 1.916205 |
| country25 | 2.26548 | 1.630329 | 1.14 | 0.257 | 0.5494926 | 9.340254 |
| country26 | 0.3393135 | 0.2045543 | -1.79 | 0.074 | 0.1035726 | 1.111624 |
| country27 | 0.562932 | 0.3819395 | -0.85 | 0.398 | 0.1480612 | 2.14028 |
| country28 | 1.283686 | 0.8433021 | 0.38 | 0.704 | 0.352259 | 4.677951 |
| country29 | 1.047749 | 0.7540016 | 0.06 | 0.948 | 0.2541317 | 4.319721 |
| country30 | 0.9059847 | 0.5386414 | -0.17 | 0.868 | 0.2811051 | 2.919933 |
| _cons | 0.00003 | 0.0000176 | -17.72 | 0.000 | 9.44e-06 | 0.0000954 |
